# CT angiography for the assessment of EVAR complications: a pictorial review

**DOI:** 10.1186/s13244-021-01112-4

**Published:** 2022-01-15

**Authors:** Cecilia Gozzo, Giovanni Caruana, Roberto Cannella, Arduino Farina, Dario Giambelluca, Ettore Dinoto, Federica Vernuccio, Antonio Basile, Massimo Midiri

**Affiliations:** 1 Radiodiagnostic and Radiotherapy Unit, Department of Medical and Surgical Sciences and Advanced Technologies “GF Ingrassia”. Catania, Italy, Via Santa Sofia 78, 95123 Catania, Italy; 2 Neuroradiology Section, Department of Radiology (IDI), Hospital Universitari Vall d’Hebron, Universitat Autònoma de Barcelona, Pg. Vall d’Hebron 119-129, 08035 Barcelona, Spain; 3 Section of Radiology - BiND, University Hospital “Paolo Giaccone”, University of Palermo, Via del Vespro 129, 90127 Palermo, Italy; 4 Department of Health Promotion, Mother and Child Care, Internal Medicine and Medical Specialties (PROMISE), University of Palermo, 90127 Palermo, Italy; 5 Vascular Surgery Unit ARNAS Civico Di Cristina Benfratelli, Palermo, Italy; 6 Section of Radiology, Asp Siracusa, Ospedale Umberto I, Via Giuseppe Testaferrata 1, Siracusa, SR Italy; 7 Vascular Surgery Unit AOUP Policlinico ‘P. Giaccone’, Palermo, Italy

**Keywords:** Aortic aneurysm, Abdominal, Computed tomography angiography, Endovascular procedures, Blood vessel prosthesis implantation, Stents

## Abstract

Endovascular aneurysm repair (EVAR) is a minimally invasive treatment proposed as an alternative to open repair in patients with abdominal aortic aneurysms. EVAR consists in a stent-graft placement within the aorta in order to exclude the aneurysm from arterial circulation and reduce the risk of rupture. Knowledge of the various types of devices is mandatory because some stents/grafts are more frequently associated with complications. CT angiography is the gold standard diagnostic technique for preprocedural planning and postprocedural surveillance. EVAR needs long-term follow-up due to the high rate of complications. Complications can be divided in endograft device-related and systemic complications. The purpose of this article is to review the CT imaging findings of EVAR complications and the key features for the diagnosis.

## Key points

- The rate of complications after endovascular aneurysm repair (EVAR) is 16–30%.
- CT angiography is the reference standard for postprocedural surveillance of EVAR.
- Knowledge of implanted device allows adequate interpretation of post-EVAR CT angiography.
- Complications after EVAR can be divided in endograft device-related and systemic.
- The most common endograft device-related complications are endoleak and device migration.

## Introduction

Endovascular aneurysm repair (EVAR) is a minimally invasive procedure introduced as an alternative to open repair (OR) in patients with abdominal aortic aneurysms (AAAs) [1]. EVAR consists of placing a stent-graft within the aorta to exclude the aneurysm from arterial circulation and reduce the risk of rupture [2]. Compared to OR, EVAR has a lower operative mortality and a shorter hospital stay [1]. Conversely, OR is known to be more durable and the repair is likely to last for the rest of the patient’s lifetime [1]. Since EVAR has an early survival benefit but an inferior prolonged survival benefit compared to OR, it needs long-term post-repair surveillance and possible reintervention to correct graft-related complications [1]. CT angiography (CTA) plays a crucial role for both preprocedural planning and postprocedural surveillance [2, 3]. The rate of complication after EVAR is high, ranging between 16 and 30% [4]. Complications can be divided in endograft device-related and systemic complications [3], as summarized in Table 1. Concerning the degree of severity, a non-standardized classification of complication in “minor,” “moderate” and “severe” one, can be replaced by a standardized and reproducible CIRSE (Cardiovascular and Interventional Radiological Society of Europe) complication classification system [5, 6].

**Table 1:** EVAR complication and related CT findings

Complications	Type of complication	CT findings	Incidence
Endograft device-related	Major suture breaks and metal-ring fractures	Discontinuity of suture points and/or metallic frame	5.5%
	Endoleak	Contrast extravasation in the aneurysm sac	15–30%
	*Type I (attachment site leak)*	Centrally located, close to an attachment site of the endograft	
	*Type II (retrograde blood flow from aortic branches)*	Peripherally located, close to the origin of the involved vessels	
	*Type III (device failure)*	Centrally located, not immediately close to the attachment sites of the endograft	
	*Type IV* (*graft porosity*)	Hazy opacification around the stent-graft, without detectable sources of endoleak	
	*Type V* (*endotension*)	Expansion of the aneurysm sac without signs contrast extravasation	
	Device migration	Device movement: > 10 mm on the centerline or > 15 mm on either the anterior or posterior aortic margin	1–10%
	Device kinking	Device angulation more frequently localized at stent-graft limb	2–4%
	Graft thrombosis and occlusion	Non-enhancing concentric or eccentric thrombus along the internal wall of the endograft	0.5–11%
	Infection	Mesenteric fat stranding adjacent to the stent-graft, perigraft fluid collections, abnormal enhancement, air bubbles and erosion into adjacent structures	0.4–3%
	Access site complication	Pseudoaneurysm: tear of the arterial wall with a blood collection, contained by the adventitia or by the surrounding perivascular soft tissues; thrombosis; dissection; hematoma; infection; lymphocele	3–5%
Systemic	Ischemia	Limb ischemia	9%
		Bowel ischemia	
		Spinal cord ischemia	

The purpose of this article is to review the CT imaging findings of EVAR complications and the key features for the diagnosis. We firstly describe EVAR eligibility, CT angiography technique and the different types of devices used for EVAR and then discuss the various types of endograft device-related and systemic complications.

## Evar eligibility

The indication for AAA treatment (surgical or EVAR) includes aneurysm diameter > 5.0–5.5 cm or symptomatic, and an increase in aneurysm size > 5 mm in a 6-month interval and > 10 mm per year [3, 7].

The choice of EVAR instead of OR depends on both patient’s contraindication to surgery and aneurysm characteristics [8].

Patients ≥ 80 years old, obese, diabetic, with cardiac, pulmonary or renal disfunction, and American Society of Anesthesiologists (ASA) III/IV are considered at high risk for surgery and may be eligible for EVAR [8, 9].

Preprocedural CT angiography allows to define aneurysm morphology and preprocedural planning.

The aneurysm anatomical characteristics suitable for EVAR include aneurysm sac diameter < 7 cm, iliac artery angulation > 90° (< 90° without diffuse calcification) and external iliac artery diameter > 7 mm and < 14 mm [9]. To emphasize the importance of proximal neck evaluation, an “aortic neck scoring system” was introduced in order to stratify the risk of graft failure [10].

This score considers proximal neck length, diameter, angulation and the amount of calcification and thrombus, which must be, in an ideal situation, ≥ 15 mm of lenght, < 30 mm in diameter, > 120° of angulation, and with wall calcification extending to less than half of its circumference without significant thrombus apposition, respectively [9, 10].

EVAR contraindications include, in addition to the lack of the above-mentioned criteria, aneurysm involving both iliac arteries, or a hypogastric artery in the case of contralateral occlusion, Marfan syndrome or acute inflammatory AAA [9].

## CT angiography technique

CTA is considered the reference standard for both preprocedural planning and postprocedural surveillance [2, 3]. Preprocedural CTA imaging allows to define aneurysm size and morphology, proximal and distal landing zones and access vessel assessment [7]. Postprocedural surveillance allows confident assessment of endograft device-related and systemic complications both early and late ones [7].

A triphasic CTA study is typically performed before and after the intravenous injection of a 90–130 ml bolus of iodinated high-concentration contrast medium through an automated injector (flow rate of 3–5 ml/s, and, sometimes, even higher) in an antecubital vein [11].

The CTA protocol includes a *non-contrast acquisition* to differentiate calcifications from contrast leakage, an *early arterial phase* (12 s after the bolus-tracking threshold) to detect most of endoleaks, and a *delayed phase* (120–300 s) to detect low-flow endoleaks (usually type II endoleaks) [3, 12, 13].

CTA allows postprocessing reconstructions such as maximum intensity projection (MIP), curvilinear reformation (CVR) and volume rendering (VR), that facilitate the detection of complications [11].

Considering the requirement of a lifelong surveillance, patients treated with EVAR may absorb a substantial cumulative radiation dose [14]. Current guidelines recommend as time interval of follow-up, CTA at 1 month, 6 months, and 12 months or whenever complications are suspected [3, 15, 16]. Therefore, for radioprotection purposes, the acquisition of non-enhanced CT scan (NECT) is generally avoided after the first CT follow-up. New generation dual-energy CT (DECT) scanners can obtain unenhanced images by subtracting iodine density from contrast-enhanced acquisitions without the need of a separate NECT scan [5, 17].

DECT angiography could be used as a single-phase examination with lower radiation exposure to the patient compared to the standard triple-phase CTA [17]. Although DECT has the advantage of a higher contrast-to-noise ratio of iodine in the blood which improves the detection of endoleaks, virtual non-contrast images may reduce the perceived visibility of calcifications [5, 17, 18].

Other diagnostic techniques include digital subtraction angiography (DSA), magnetic resonance angiography (MRA) and contrast-enhanced ultrasound (CEUS) [19]. DSA is an invasive procedure currently reserved for preprocedural planning or intraprocedural guidance [3]. Contrast-enhanced-MRA with gadolinium may be useful in patients with contraindications to iodinated contrast administration (i.e., iodine allergy) [3]. MRA has the advantage of the possibility to avoid the use of contrast medium, with techniques such as time-of-flight magnetic resonance angiography (TOF-MRA) [3, 19].

CEUS is a minimally invasive modality in detection of complications, cost-effective, with lack of ionizing radiation use, but difficult to reproduce [19].

## Type of device

**Fig. 1:**
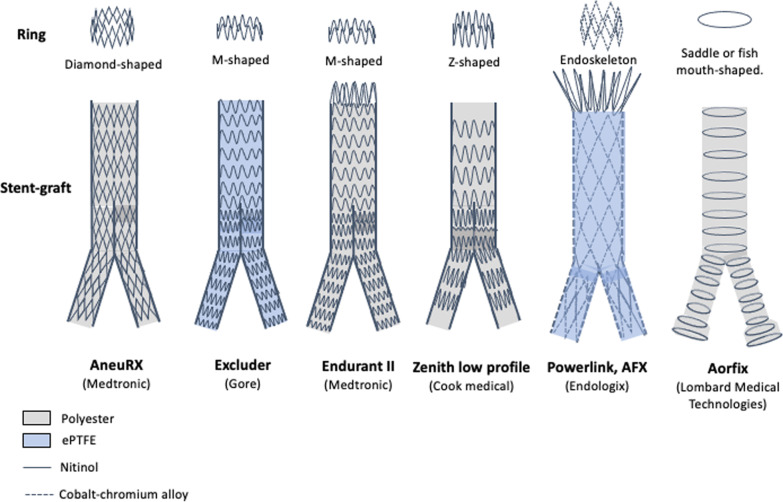
Drawings illustrating the various types of metal ring and stent-graft

Figure 1 depicts the various types of metal ring and stent-grafts used for EVAR. In regard of the material, the device is formed by a metal-structure (i.e., stainless steel nitinol or cobalt-chromium alloys), while the graft is composed of polyester or polytetrafluoroethylene (ePTFE) fabric [20, 21]. Based on the anatomical structure, stent-grafts can be divided in bifurcated (aorto-bi-iliac) or aorto-uni-iliac [20]. Stent-grafts usually adopt a modular design with at least two component grafts: in detail two (bimodular) or three (trimodular) separate components, including an aortic bifurcated main body and one or two iliac limbs [20, 22]. More rarely, some stent-graft can be unibody (e.g., *Powerlink*) [20]. Depending on the level of fixation relative to the renal arteries, they are also subdivided into suprarenal (proximal to renal arteries, e.g., *Zenith low profile*, *Endurant II, Aorfix*) or infrarenal (distal to renal arteries, e.g., *Excluder*) [20, 21].

The most common and old type is the *AneuRx* (Medtronic, Santa Rosa, Calif) formed by nitinol stent rings and woven polyester graft material; each stent ring is a series of diamond-shaped segments connected side to side [23]. The *C3 Excluder* (W. L. Gore & Associates, Flagstaff, Ariz) is a modular bifurcated system composed of PTFE approved for infrarenal necks measuring ≥ 15 mm in length and ≤ 60 degrees angulation [24]. The *Endurant II* (Medtronic, Santa Rosa, CA) is a modular bifurcated stent-graft composed of M-shaped nitinol stents and polyester graft. It is approved for infrarenal necks of ≥ 10 mm length with angulation ≤ 60 degrees or of 15 mm and ≤ 75 degrees [25]. The *Zenith low profile* (Cook Medical, Bloomington, USA) is a modular bifurcated system consisting of a nitinol Z-stents sutured to a woven polyester graft material. The proximal stent contains barbs for suprarenal fixation. It is approved for a minimum 15 mm infrarenal neck and an infrarenal angulation of ≤ 60 degrees [21].

The *AFX* stent-graft, as well as its predecessor *Powerlink* (Endologix, Inc. Irvine, Calif), is a unibody stent-graft consisting of an inner endoskeleton with multiple metallic struts of cobalt–chromium alloy covered by a thin-walled ePTFE-graft fabric outer cover; these two stent-graft layers are sutured only at the proximal and distal ends [26, 27]. The *Aorfix* stent-graft (Lombard Medical Technologies, Oxfordshire, UK) is composed of saddle or fish mouth-shaped nitinol rings on a polyester fabric. This shape allows the rings to be placed trans-renally with the fish mouth trough aligned with the renal arteries juxta-renally and the fish mouth peak extending suprarenally. It is approved for a neck length of ≥ 20 mm, and it is the only available endograft that can be used in infrarenal angulation up to 90° [25].

Although EVAR was initially limited to aneurysms with a neck long enough to accommodate the stent-graft, for AAAs with a short or absent neck involving visceral arteries (superior mesenteric artery, right renal artery or left renal artery), a fenestrated and branched stent-grafts (*f/b EVAR*) are proposed [28].

## Evar complication classification

EVAR complications can be classified by time of onset, cause and severity.

Based on the time of appearance, they can be divided into immediate (days 0–1), early (days 2–30) and late complications (days 31 +) [29].

Regarding the cause, complications can be divided in endograft device-related and systemic complications [3], as summarized in Table 1.

Concerning the degree of severity, a non-standardized classification divides complications in “minor,” “moderate” and “severe” ones [5]. In 2017, CIRSE Standards of Practice committee introduced a standardized and reproducible system for the classification of complications consisting in a grading scale from one to six where grade “one” is assigned to a complication that may be solved within the procedure operative session without additional therapy, sequelae or deviation from the normal post-therapeutic course, and “six” is assigned in case of death [6].

## Endograft device-related complications

Endograft device-related complications occur in 16–30% of patients after EVAR; the most common types include endoleak (occurring in 15–30%) followed by device migration (1–10%), graft limb thrombosis (0.5–11%), and structural endograft failure (5.5%) [3, 4, 30, 31].

### Endoleak

Endoleak (EL) is considered the most frequent complication after EVAR, occurring in 15–30% of patients within the first 30 postoperative days [3]. EL is defined as the persistent perigraft blood flow within the aneurysm sac with contrast opacification changing in degree and shape between the arterial and delayed phases [32, 33]. ELs are classified in five types (Table 1) according to the origin of the blood flow [34].

**Fig. 2:**
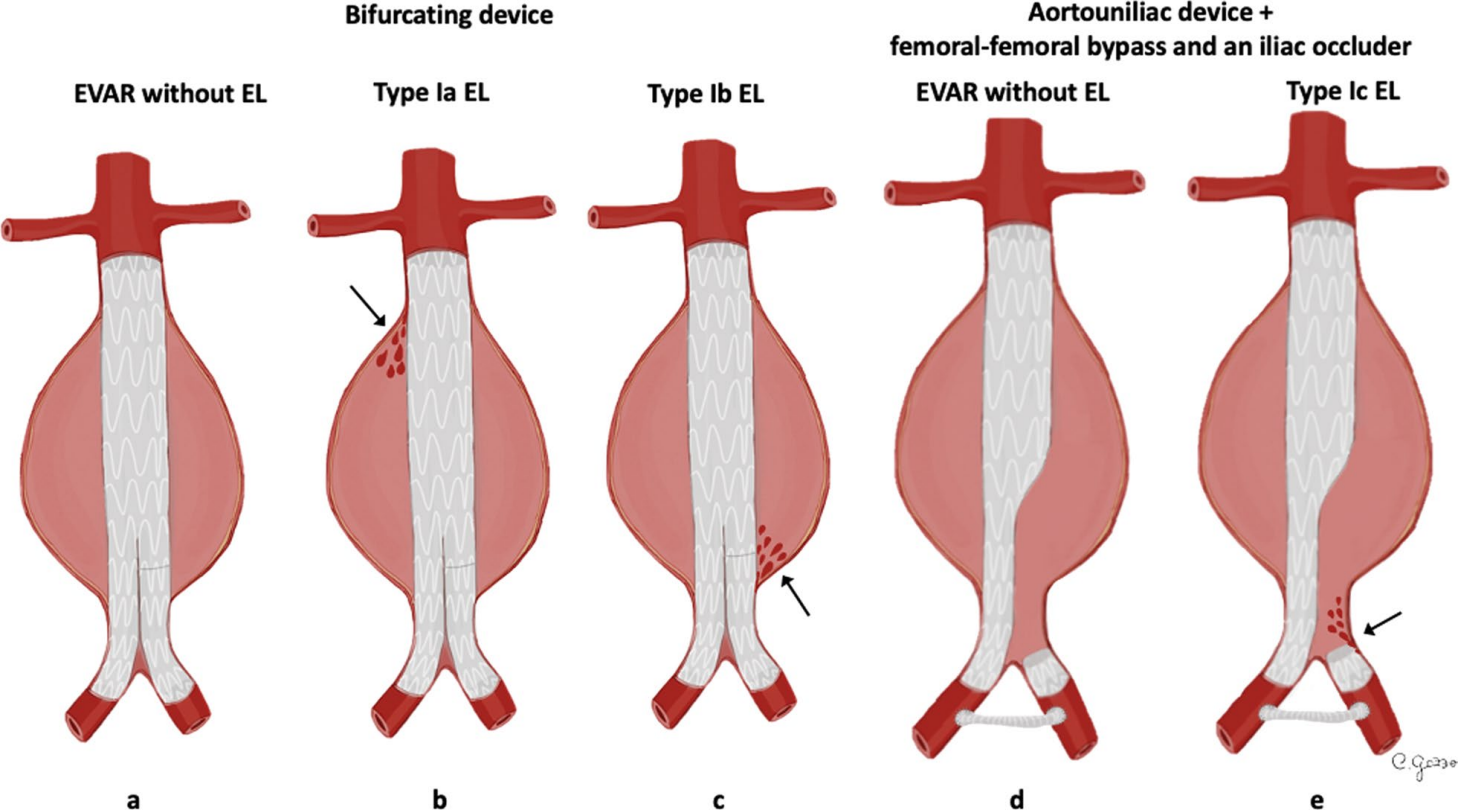
Drawings illustrating the physiopathology of *type I* endoleak. **a** A typical EVAR device consisting of a main body for the aorta and one “limb” for each common iliac artery; **b** *type Ia* endoleak; **c** *type Ib* endoleak; **d** EVAR with an aorto-uniliac device, a femoral-femoral bypass and an iliac occluder; **e** *type Ic* endoleak with failure of the iliac occluder, resulting in retrograde blood flow through the common iliac artery and the aneurysm sac

**Fig. 3:**
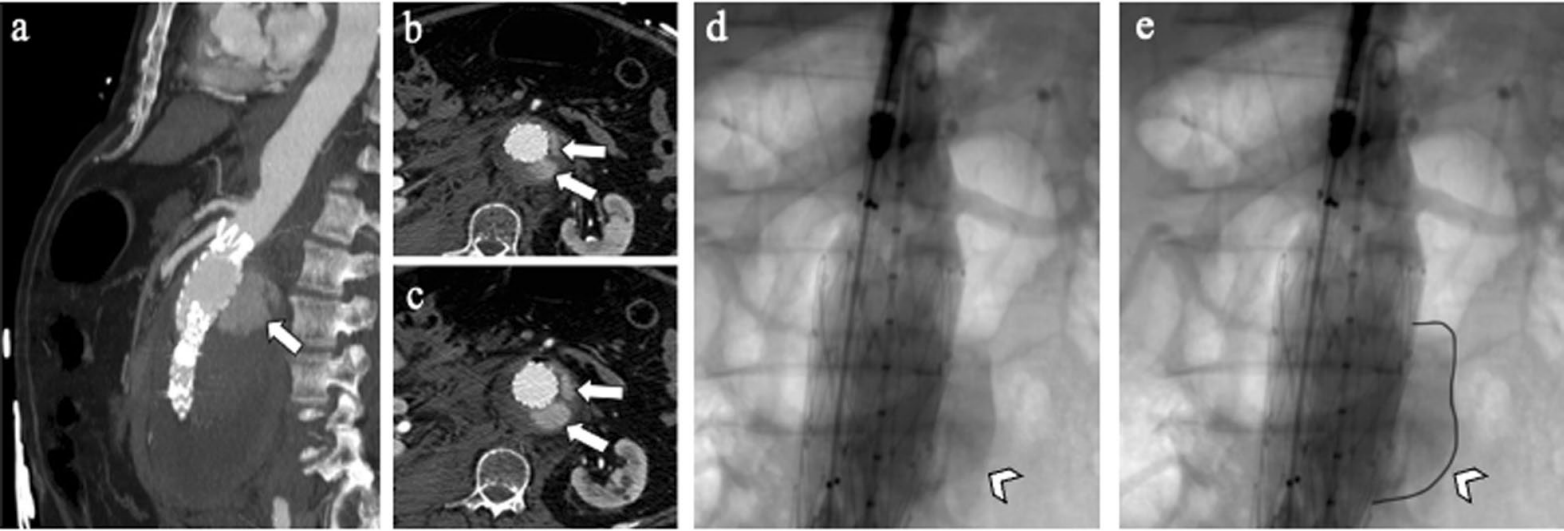
**a** Sagittal maximum intensity projection and (**b, c**) axial CT angiography images at different levels show *type Ia* endoleak in a 67-year-old man detected as contrast agent extravasation outside stent-graft within aneurysm sac (arrows), close to the proximal attachment site of the endograft; **d** intraoperative aortography performed by the pigtail catheter with tip positioned above the proximal endograft fixation shows a *type Ia* endoleak (arrowhead) in a 82-year-old man; **e** the same angiographic image shows a *type Ia* endoleak profile remarked with a black line

*Type I EL* is considered a leakage from the attachment sites of the stent-graft and native artery [33]. *Type I EL* (Fig. 2) can be divided into *type Ia* (involving the proximal attachment site, Fig. 3), *Ib* (involving the distal attachment site) and *Ic* (involving the iliac occluder) [32]. *Type Ic EL* refers to the failure of occlusion of the contralateral common iliac artery in patients with aorto-uniliac endograft placed in conjunction with a femoral-femoral bypass. *Type I EL* is more frequent in patients with complex arterial anatomy: short, angulated, or tapered proximal necks leading to an imperfect seal between the stent-graft and the aortic walls, resulting in *type Ia EL* [32]. In the same way, dilated, irregular and tortuous iliac arteries increase the risk of *type Ib EL* [27, 35]. CT scan may show the presence of contrast agent extravasation more pronounced in the central part of the aneurysm sac, close to an attachment site of the endograft. Due to the direct communication with the aortic blood flow, *Type I ELs* tend to evolve and usually need to be treated [32]. Endovascular repair for *Type I EL* is successful in acute and asymptomatic presentation, but surgical conversion must always be considered if endovascular approach is unsuccessful [36].

**Fig. 4:**
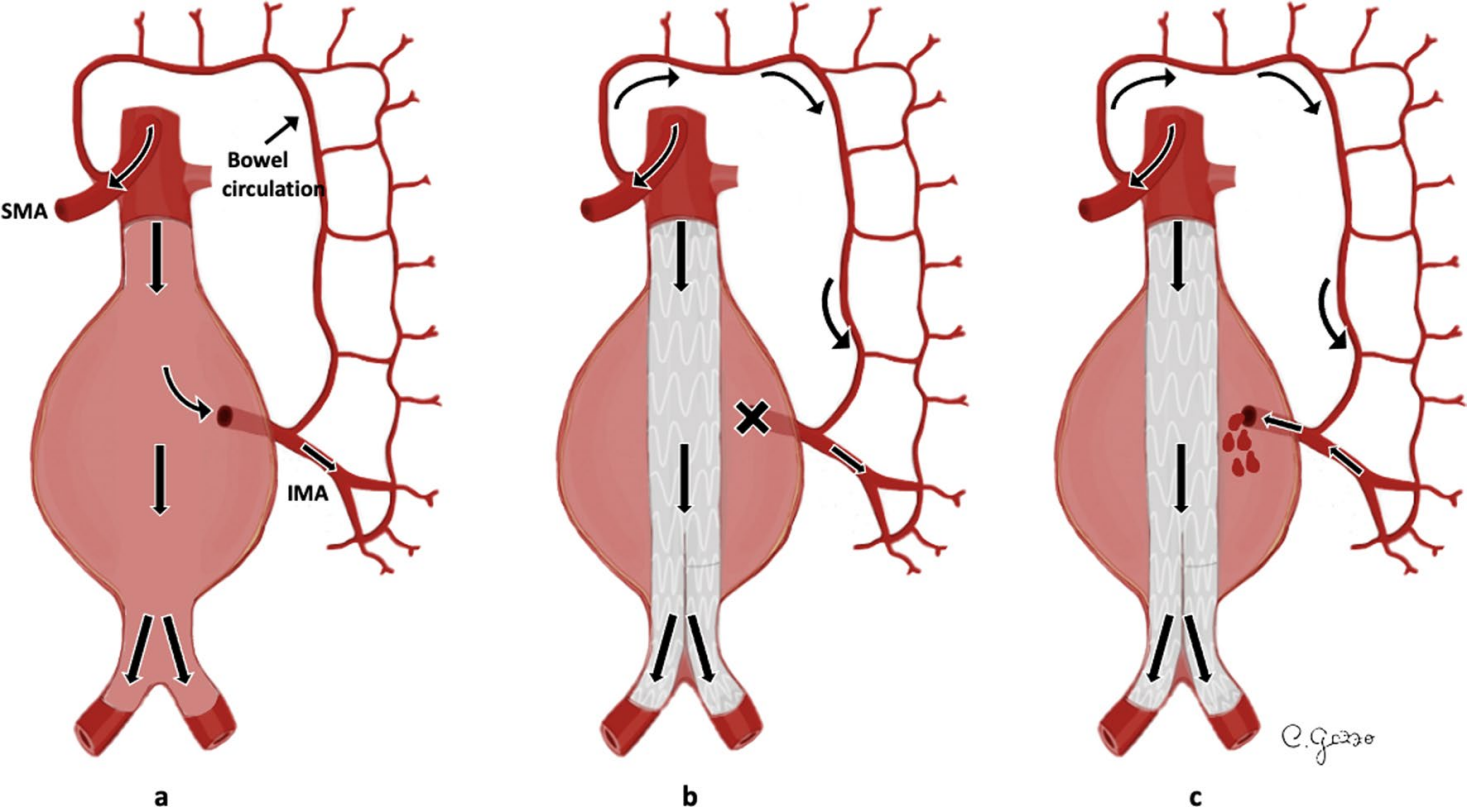
Drawing illustrating the physiopathology of *type II* endoleak. **a** Schematic representation of an abdominal aortic aneurysm (AAA) with a normal bowel circulation, supplied by superior mesenteric artery (SMA) and inferior mesenteric artery (IMA); **b** blood circulation after a typical EVAR: normal flow in the SMA, that provides blood to the major part of the bowel through collateral circles shared with the territories irrigated by the IMA; no flow in the IMA, that originates from the excluded aneurysm sac; **c** *type II* endoleak: collateral vessels provide retrograde blood flow to the IMA, bringing blood inside the aneurysm sac

**Fig. 5:**
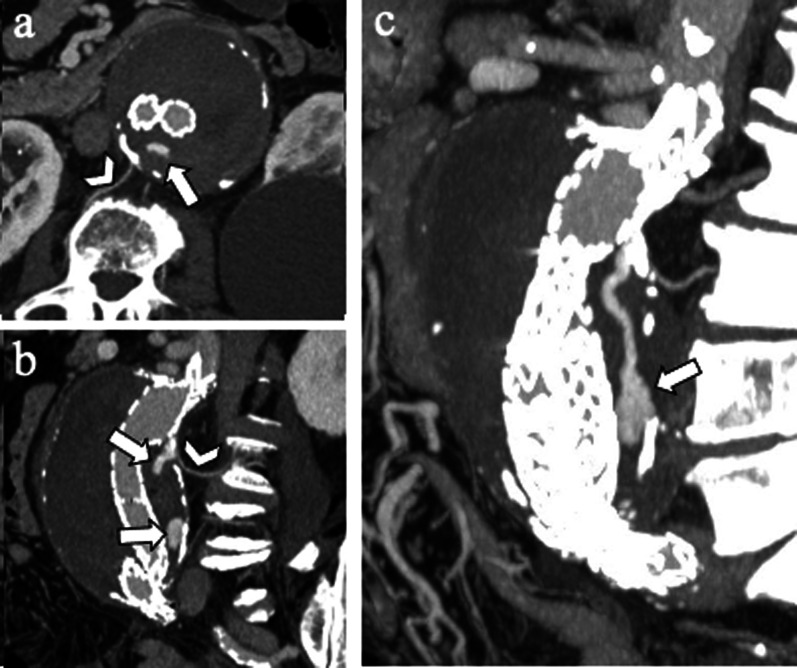
86-year-old man with *type IIa* endoleak. **a** Axial and (**b, c**) sagittal maximum intensity projection CT angiography images show *type II* endoleak (arrows) due to retrograde perfusion of a lumbar artery (arrowheads)

*Type II EL* is the most common type of endoleak with a reported prevalence between 8 and 44% [36]. In *type II EL*, the persistent perfusion of the aneurysm sac is caused by retrograde blood flow via collateral vessels (most commonly the inferior mesenteric artery and lumbar arteries) [38]. Based on the number of patent branches involved, *type II EL* (Fig. 4) can be classified into *type IIa* (only one collateral artery, Fig. 5) and *type IIb* (two or more arteries) [31]. Up to 40% of *type II EL* spontaneously resolve with collateral thrombosis [30]. Therefore, the “wait and see” approach is accepted for stable aneurysms at follow-up [32, 38]. *Type II EL* treatment is advisable for cases persistent over 6 months and with more than 5 mm of sac expansion compared to preprocedural CT measurements [39]. CT scan shows the presence of contrast agent in the peripheral part of the aneurysm sac, close to the origin of the involved vessels (anterior wall of the sac for inferior mesenteric artery, posterior wall of the sac for lumbar arteries). Minimally invasive treatment includes embolization through transarterial, transcaval, translumbar and transabdominal approaches. The translumbar embolization is a safe and effective treatment for *type II EL*, with low complication and reintervention rate [38].

**Fig. 6:**
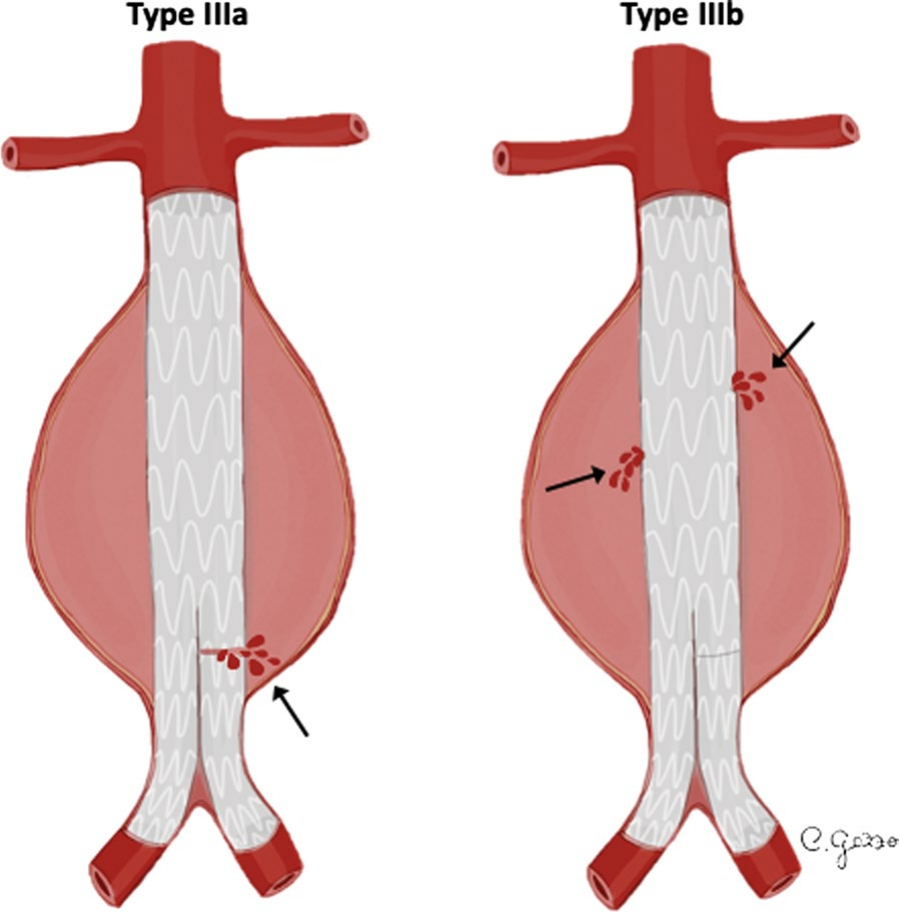
Drawing illustrating the physiopathology of type III endoleak. From left to right*: type IIIa* endoleak and *type IIIb* endoleak are characterized, respectively, by disconnection between modular components (*IIIa*) and structural stent-graft failure (*IIIb*)

**Fig. 7:**
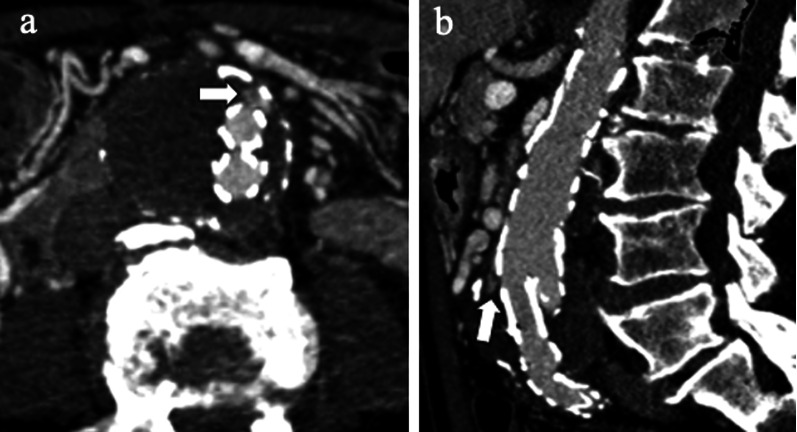
82-year-old man with *type IIIa* endoleak. **a** Axial and (**b**) sagittal CT angiography images show contrast agent outside stent-graft (arrows) close to the junction of the main body with the iliac limb

**Fig. 8:**
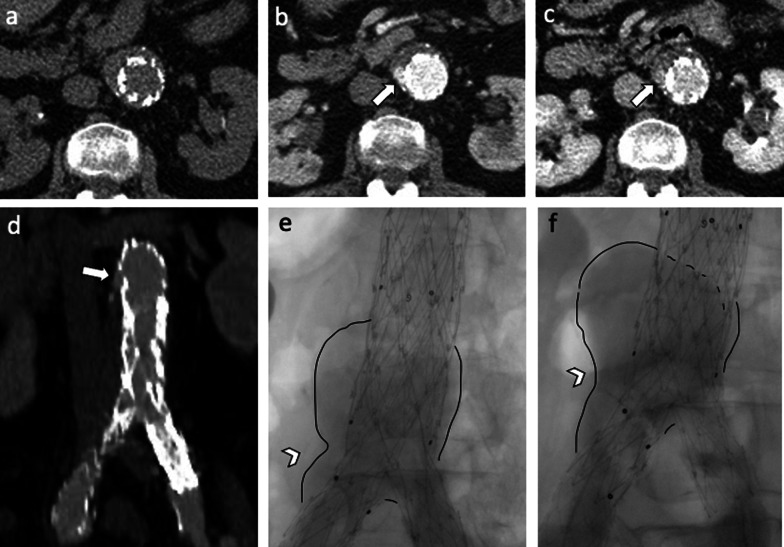
67-year-old woman with *type IIIb* endoleak: Axial CT images on (**a**) pre-contrast, (**b**) arterial and (**c**) delayed phases and (**d**) coronal arterial phase show a type IIIb endoleak (arrows); intraoperative aortography in (**e**) anteroposterior and (**f**) caudo-cranial projections performed in the same patient confirmed endoleak (arrowheads) with profile remarked through a black line

*Type III EL* is a rare complication (incidence of 4% beyond 1 year) attributed to structural stent-graft failure or disconnection between modular components (Fig. 6) [32]. *Type III EL* can be divided into *IIIa* (junctional separation of modular components of the device) (Fig. 7) and *IIIb* (stent-graft fabric disruption, Fig. 8) [32, 40]. CT scan shows the presence of contrast extravasation at the central of the aneurysm sac, adjacent to the endograft but not immediately close to its attachment sites (may be close to the junction of modular components in case of *type IIIa* endoleak). As *type I EL*, *type III EL*s are considered high-pressure, high-risk leaks and always warrant urgent management [32]. Treatment of *type III EL* includes endovascular approach with placement of a covered stent across the gap between the original stent-graft components or across the fabric disruption [40].

**Fig. 9:**
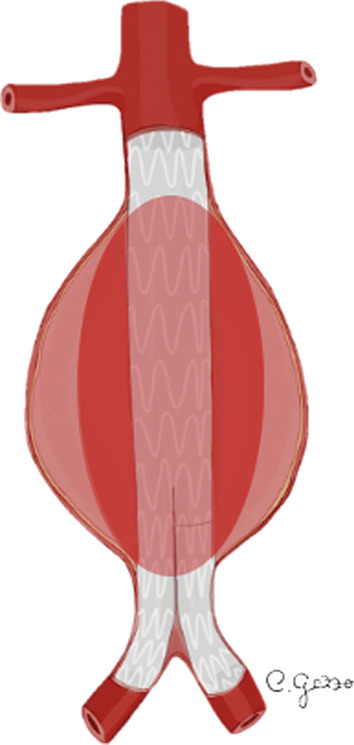
Drawing illustrating the physiopathology of *type IV* endoleak

*Type IV EL* is a complication caused by porosity of the endograft fabric, which can be angiographically seen during the placement of the device or immediately after (Fig. 9) [34]. *Type IV EL* is related to the anticoagulant treatment of the patient and it spontaneously resolves within 30 days [32]. It is uncommon with new generation devices. Angiographic study shows hazy opacification around the stent-graft, without detectable sources of EL.

**Fig. 10:**
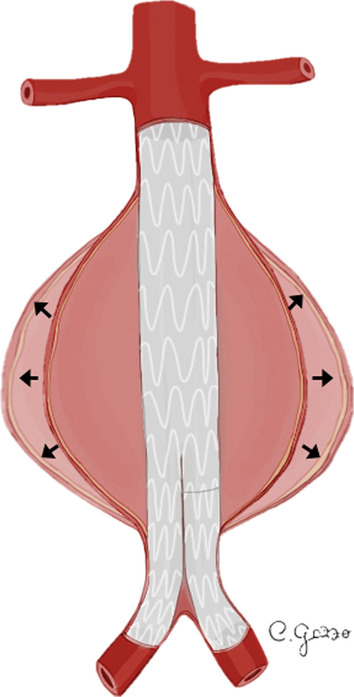
Drawing illustrating the physiopathology of *type V* endoleak. Expansion of the aneurysm sac observed in follow-up examination, without signs of other types of contrast extravasation

*Type V EL*, also named “*endotension*,” is referred to the expansion of the aneurysm sac without signs of other types of EL (Fig. 10) [3]. *Type V EL* is a diagnosis of exclusion, thus an occult endoleak has to be investigated using other techniques (MRA, CEUS). Other causes of endotension include ultrafiltration of blood through the stent-graft fabric, transmission of the blood pressure to the aortic wall through the thrombus around the device, infection and seroma. No specific treatment is recommended for *type IV* and *type V ELs* [3].

**Fig. 11:**
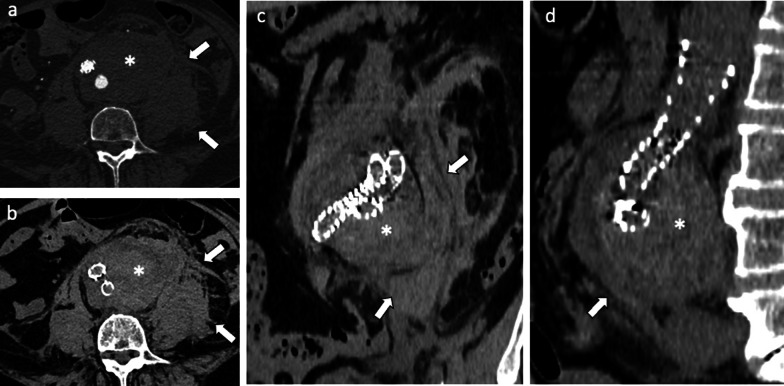
65-year-old man with *aneurismatic sac rupture*: Axial CT images on (**a**) pre-contrast, (**b**) arterial phases and (**c**) coronal and (**d**) sagittal arterial phase show left antero-lateral rupture of the aneurysm sac with periaortic hematoma (*) and blood effusion close to the left psoas muscle

The presence of EL (expecially type I) exposes to the risk of *aneurysm sac rupture* (Fig. 11) [41]*.*

In clinical practice, differential diagnosis can be difficult to establish in case of Endologix *AFX/Powerlink* stent-graft placement, due to the presence of “billowing phenomenon,” which may mimic an EL [28]. On CT scan, the billowing phenomenon appears as a slightly hyperdense rim of density beyond the endoskeleton within the outer fabric material, with typical cauliflower-like shape [42]. On CT angiography, contrary to endoleak, in Endologix stent-graft, the intravenous contrast agent stays confined inside the curvilinear thin hyperdense line of the graft cover, without any connection to the excluded aneurysm sacs, and maintains the same curvilinear configuration as before the contrast [43]. Although billowing was firstly considered a benign condition, the progression of this phenomenon may cause a continued pressurization of the AAA sac with risk of rupture [42].

### Suture breaks and metal-ring fractures

The structure of *AneuRx* stent-graft predisposes the device to two kinds of mechanical damage: metal-ring fracture and suture break [24]. At CT scan, metal-ring fracture appears as a discontinuity of a metallic frame [24]. Suture break is defined as breakage of the polyester sutures that connect adjacent rings leading to their separation and discontinuity of consecutive suture points [24]. Based on the proportion of the circumference of two adjacent metallic ring of stent involved, suture breaks can be classified as minor (< 180° of the circumference) or major (> 180° of the circumference) [24]. Particularly, major suture breaks and metallic stent-ring fractures demonstrated at CT scan are associated with delayed type I and III endoleaks and with stent-graft migration after EVAR [24].

Both suture breaks and metal-frame fractures can involve one of the seven segments of the endograft: (a) the main body, (b) the junction between the main body and the limbs of the bifurcated graft, (c) the long limb of the bifurcated graft, (d) the extender cuff attached to the long limb, (e) the short limb of the bifurcated graft, (f) secondary limb attached to the short limb in the aorta (excluding overlap with short limb), and (g) the extender cuff attached to the secondary limb [24].

**Fig. 12:**
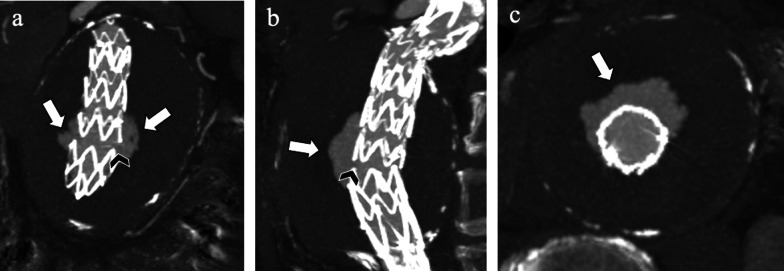
85-year-old man with a probable rupture of PTFE (polytetrafluoroethylene) graft material and *type IIIa* endoleak. **a** Coronal, (**b**) sagittal, and (**c**) axial maximum intensity projection CT angiography images show contrast agent outside stent-graft (arrows) consisting in type III endoleak caused by probable PTFE fabric erosion (arrowheads)

In other stent-graft without any suture point, e.g., the *Excluder* type (W. L Gore and Associates, Flagstaff, Ariz), a rupture of PTFE graft material as a fabric disruption could be detected and be the underlying cause of IIIa endoleaks [44] (Fig. 12).

### Device migration

Device migration is defined as device movement of more than 10 mm on the centerline or movement of more than 15 mm on either the anterior or posterior aortic margin [3, 24]. Some authors considered device migration as a change of > 10 mm in the distance between the reference vessel (celiac axis or superior mesenteric artery) and the first visible portion of the stent-graft on a sagittal multiplanar CT reconstruction [45].

Device migration can affect both the proximal and distal fixation of stent-graft [46]. Changes of width and length of the aneurysm sac, hemodynamic forces and inadequate overlap between the device and the aneurysm neck can lead to device migration [47]. Stent-graft migration can be the underlying cause of *type I EL* (since the attachment sites of the endograft can be moved to a section of the vessel that do not correspond to the size and the shape of the device), *type III EL* (because of rupture of the device or disjunction of modular components) and device kinking [46–48]. Migration of the iliac limb can lead to *type Ib EL* and *type III EL* [46]. The risk factors potentially influencing limb migration include a large aneurysm (> 6 cm), dilated or aneurysmal common iliac artery (> 18 mm), short length of fixation (< 70%), and lower degree of iliac limb oversizing (< 10–20%) [46].

**Fig. 13:**
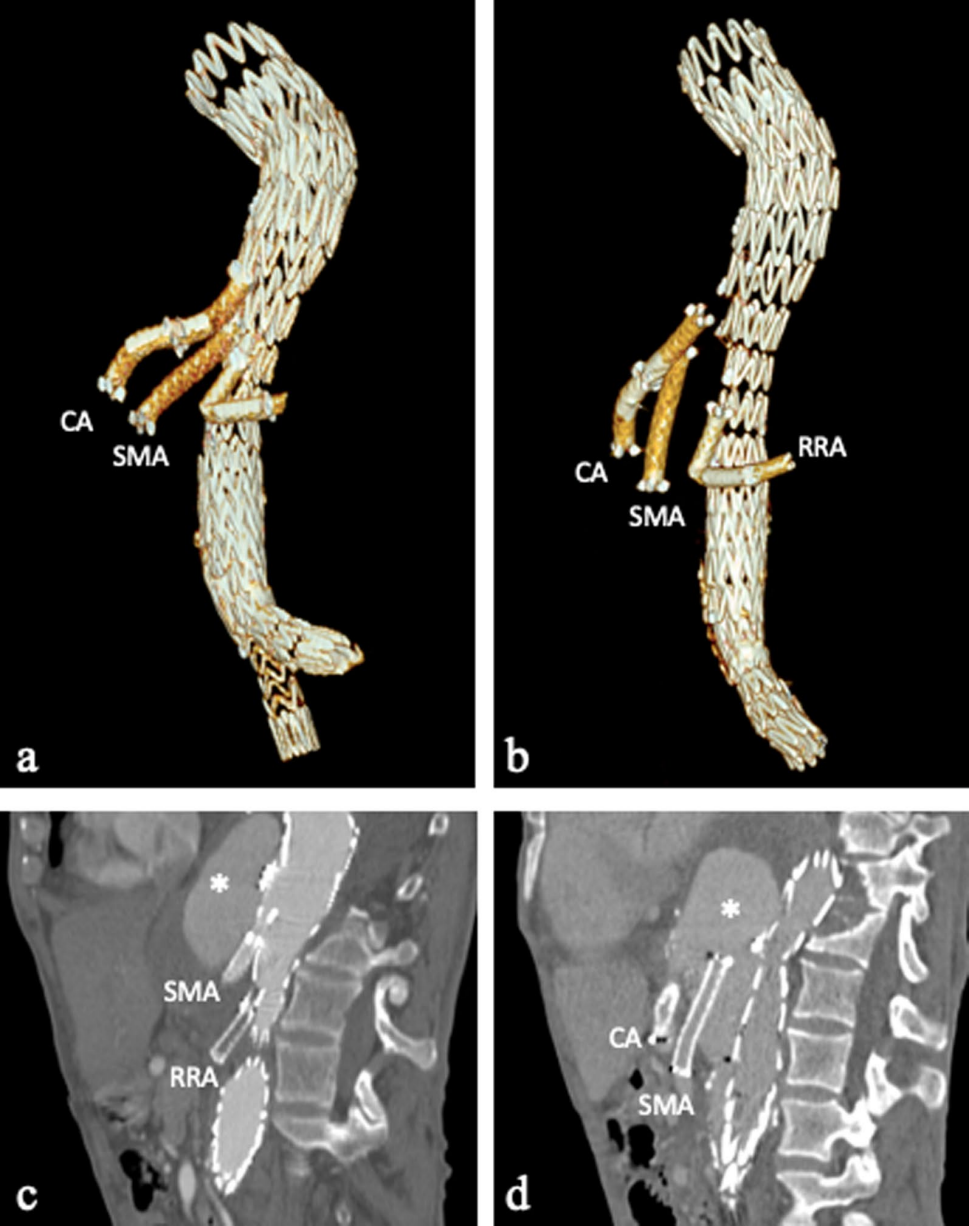
78-year-old man with stents migration. **a** Volume rendering reconstruction and **b** sagittal oblique CT images show a thoracoabdominal aneurysm treated with a f/b EVAR and three stent-grafts placement in aortic branches: celiac artery (CA), superior mesenteric artery (SMA) and right renal artery (RRA); the last one appears occluded. **c** Volume rendering and (**d**) sagittal oblique CT images after 3 years show distal migration of all three stent-grafts. In both examinations, there is contrast agent inside the aneurismal sac (*), likely a *type IIIb* endoleak due to imperfect junction of the branches with the main device body

In case of f/b EVAR with placement of visceral arteries stents (superior mesenteric artery, right renal artery or left renal artery), these latter could migrate (Fig. 13).

### Device kinking

**Fig. 14:**
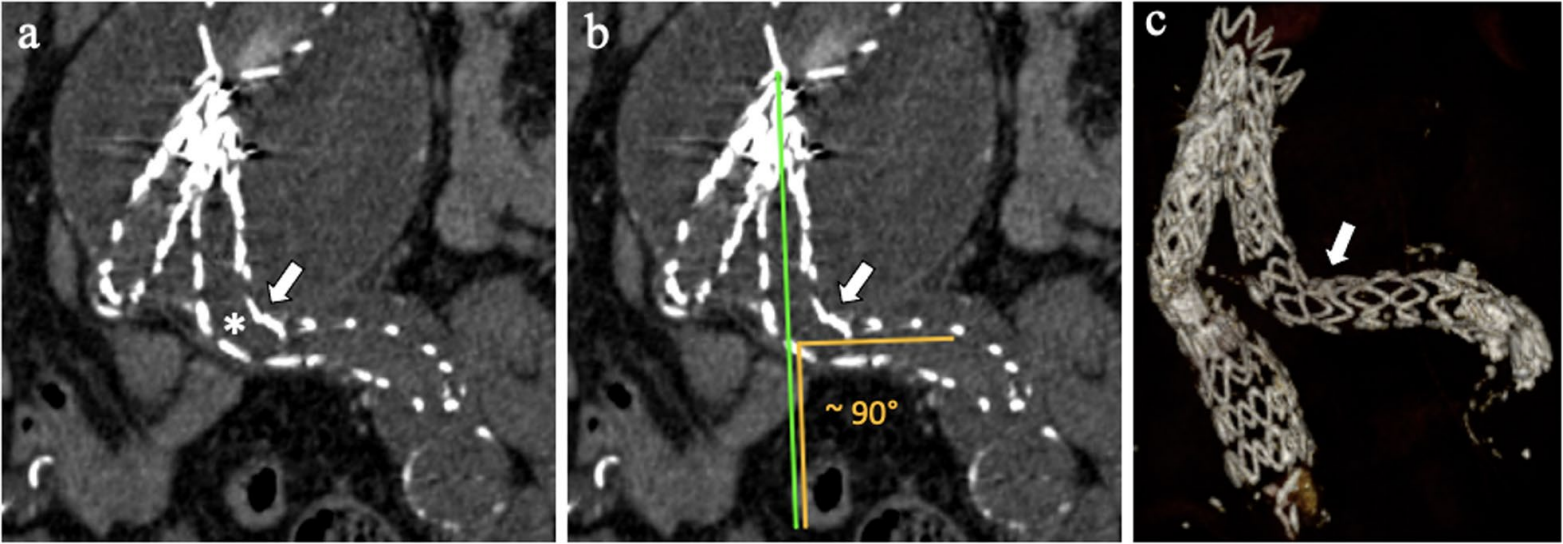
87-year-old man with left stent-graft-limb kinking. **a, b** Coronal CT images and (**c**) volume rendering reconstruction CT angiography image show left limb kinking (arrows) with angulation of about 90°, which is associated with endograft thrombosis (*)

Device kinking may occur in 2–4% of patients, due to size reduction in the residual aneurysm sac over time, severe proximal aortic neck angulation and a narrow diameter of the distal aortic neck [3, 49, 50]. Device kinking (Fig. 14) can be localized at stent-graft limb and is defined as a sharp localized angulation > 90° on radiological examination [50, 51]. Kinking can lead to device migration, *type I* and *type III EL*, endograft thrombosis and occlusion [49]. Limb kinking treatments include percutaneous angioplasty with or without placement of reinforcing stents or additional endograft limbs within the original graft [49].

### Graft thrombosis and occlusion

**Fig. 15:**
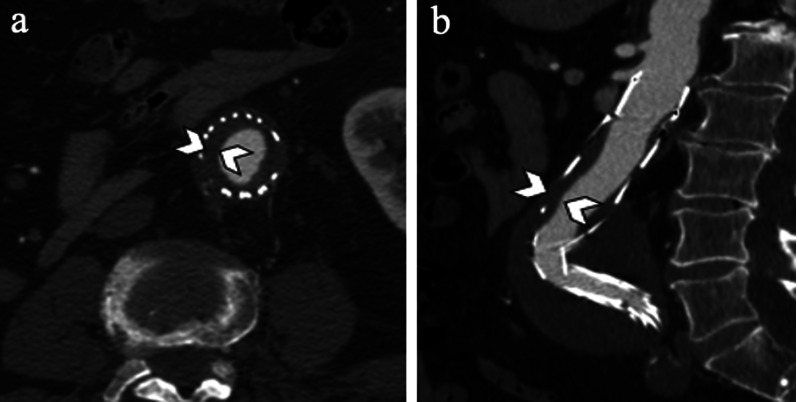
81-year-old man with stent-graft thrombosis**. a** Axial and **b** sagittal CT angiography images show non-enhancing concentric thrombus (arrowheads) inside the lumen of the endograft

Graft thrombosis is reported in 4% of patients and often related to device kinking, migration and dislocation [2, 7, 40]. Excessive oversizing can also result in folding of the graft material, with twisting of the limbs and subsequent endograft limb thrombosis [2, 40]. CT angiography scan shows a non-enhancing concentric or eccentric tissue along the internal wall of the endograft (Fig. 15). Treatment options include thrombectomy and stent placement of the thrombosed limb [2]. Sometimes, a surgical femoral-to-femoral artery bypass may be required [2].

### Infection

**Fig. 16:**
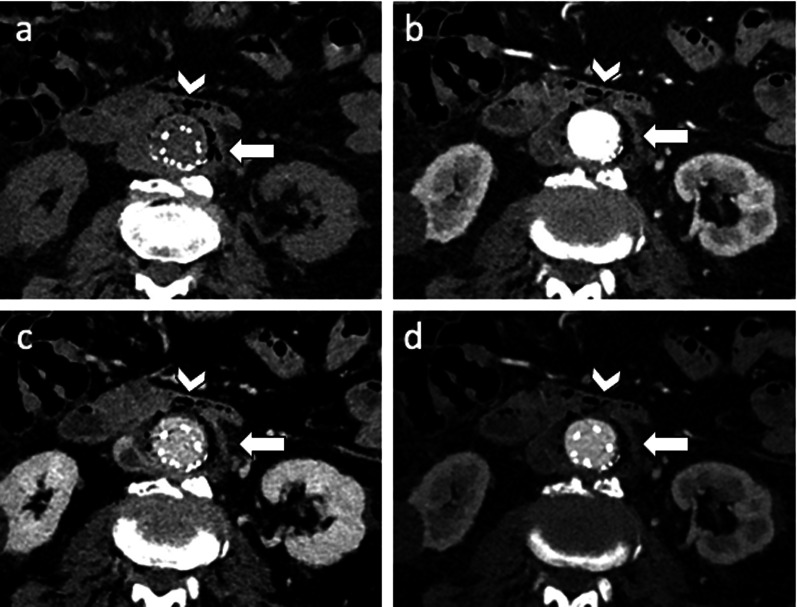
81-year-old man with aorto-enteric fistula. Axial CT images on (**a**) pre-contrast, (**b**) arterial, (**c**) portal and (**d**) delayed phases show small air bubbles around the graft (arrow) and communication between the second portion of duodenum and the aneurysm sac as an aortoduodenal fistula

Endograft infection is an uncommon complication (< 1%) with a high mortality rate [3, 7]. It is usually caused by procedural contamination or eventually by device colonization from remote sites of infection [52]. Patients with endograft infection typically show fever, leucocytosis, and back pain [3, 53]. CT scan can demonstrate periaortic fat stranding adjacent to the stent-graft, perigraft fluid collections, abnormal enhancement, air bubbles, and erosion into adjacent structures [52]. Cases of aorto-enteric fistulas (Fig. 16) have been described in the literature with significant poor prognosis [52, 54].

Endograft infections may be managed conservatively with antibiotics and possible percutaneous drainage; severe cases could require endograft removal [52, 53, 55, 56].

### Access site complications

The EVAR percutaneous procedure begins with the puncture of the femoral artery as access site which can be associated with several local complications reported in 3–5% of patients [7]. These latter can be related to patients’ predisposition (i.e., obesity or severe femoral artery calcification) or access technique (i.e., lack of operator experience, repeated groin access, large sheath size) [57, 58]. Access site complications include arterial thrombosis, pseudoaneurysm, dissection and other local wound complications (i.e., groin hematoma, lymphocele and infection) [2, 58]. In order to avoid these complications, some authors suggest preprocedural CT scans for common femoral artery calcium arcs and meticulous ultrasound-guided arterial puncture [58, 59].

**Fig. 17:**
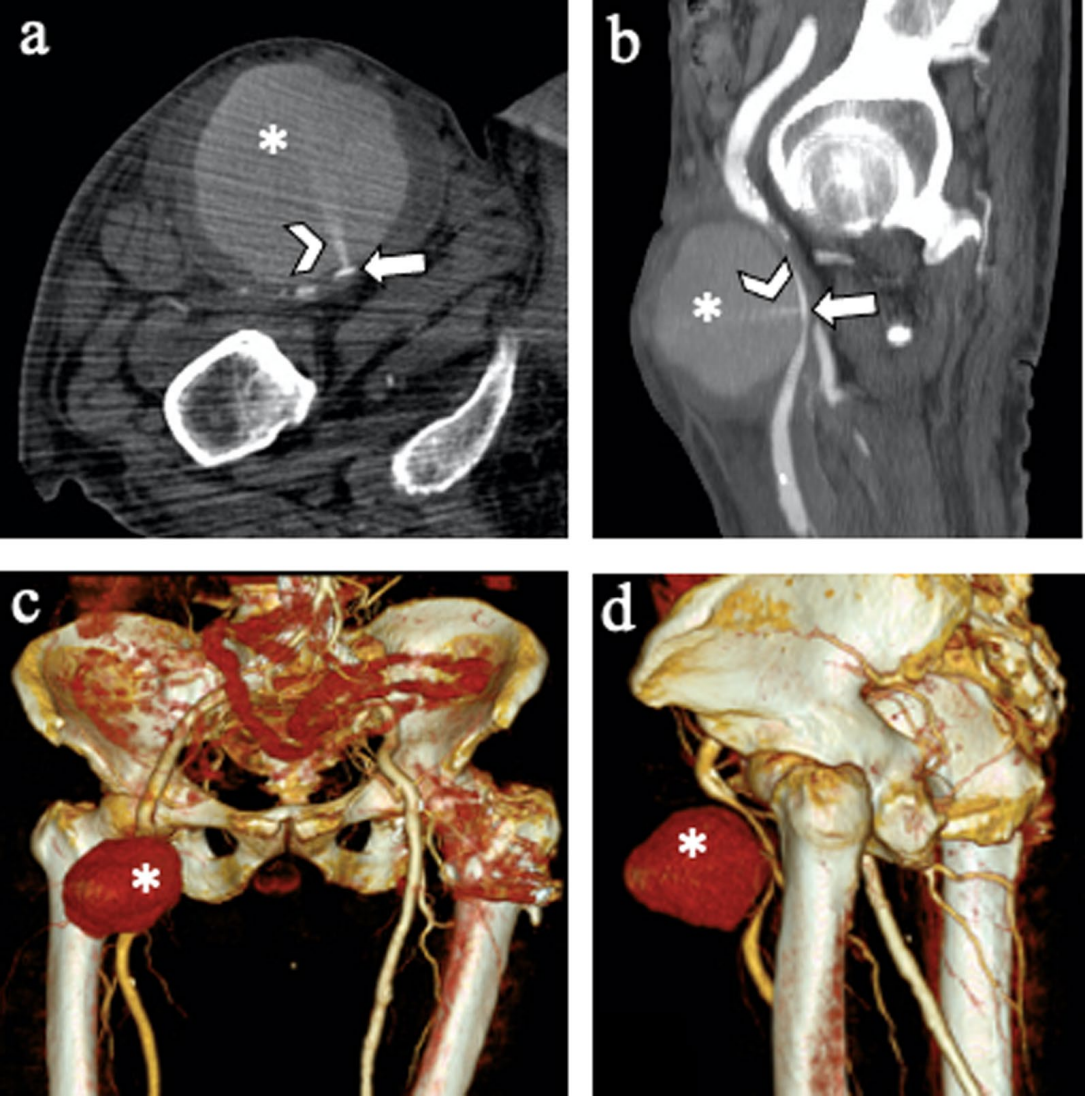
75-year-old man who underwent EVAR with right superficial femoral artery access. **a** Axial, (**b**) sagittal, and (**c, d**) volume rendering reconstruction CT angiography images show an access site complication: a pseudoaneurysm (*) of the right superficial femoral artery (arrows). It is also possible to see a contrast jet originating from the artery (arrowheads)

CT scan can show pseudoaneurysm, thrombosis, dissection and other local wound complications, i.e., hematoma, infection and lymphocele. On CT scan, pseudoaneurysm appears as a tear of the arterial wall with a hematic collection, contained by the adventitia or by the surrounding perivascular soft tissues (Fig. 17).

## Systemic complication

### Ischemia

Ischemic complications are reported in approximately 9% of patients causing by arterial thrombosis, embolism, dissection, or obstruction occurring as a result of endograft malposition [3]. It can involve the lower extremities and also the kidneys, bowel and pelvic organs.

**Fig. 18:**
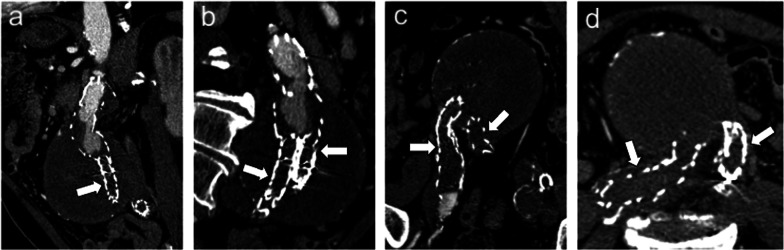
87-year-old man with bilateral iliac stent occlusion. **a, b** Sagittal and (**c**, **d**) CT multiplanar reconstruction images show non-enhancing concentric thrombus inside the lumen of the endograft limbs, with subsequent endograft limb occlusion (arrows)

*Lower Limb ischemia* is the most common type of ischemia after EVAR, and it may be the result of endograft limb occlusion [59]. Patients may complain with pain, paresthesia, intermittent claudication and decreased femoral pulse [59]. CT angiography scan is helpful to underlain the cause of the ischemia such as endograft thrombosis, occlusion (Fig. 18), and kinking.

**Fig. 19:**
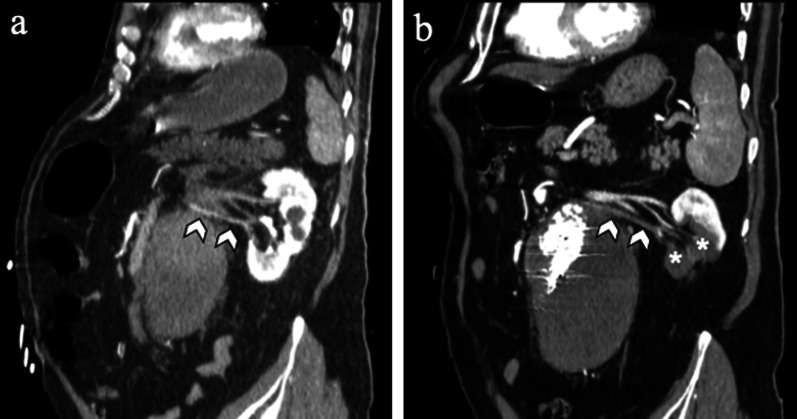
68-year-old man with segmental renal ischemia. **a** Sagittal CT angiography image shows left kidney normally perfused by the main left renal artery (not showed) and by an accessory renal artery (arrowheads); **b** sagittal CT image obtained from the same patient after EVAR shows occlusion of the accessory renal artery and lack of enhancement in the lower renal pole (*), which appears reduced in volume, due to chronic ischemia

*Renal ischemia* could be secondary to arterial thrombosis embolus, dissection, and inadvertent coverage of the origin of the renal arteries in patients with multiple accessory arteries by the endograft or endograft migration (Fig. 19) [3, 60]. The inadvertent coverage of the renal arteries by the endograft may be more frequent in case of a short aortic neck [3]. In addition to ischemia, kidney may be exposed to contrast nephropathy [61].

*Bowel ischemia* after EVAR most commonly involves the left colon. It results from embolus or endograft coverage of the inferior mesenteric artery origin. Albeit this latter occurs in all cases of EVAR, it becomes clinically relevant in case of insufficient mesenteric collateral circulation [62]. Small bowel or right colonic ischemia is less common [3].

*Pelvic ischemia* after EVAR is more frequent in case of intentional embolization of the internal iliac arteries as in the case of internal iliac artery aneurysms exclusion. Clinical signs may include buttock claudication, rectal ischemia, erectile dysfunction and possible skin necrosis [3].

*Spinal cord ischemia,* although rare, is reported in the literature as EVAR-related complication [59, 63, 64]. It typically develops within 12 h following EVAR and may cause paraplegia [3, 64]. The underlying causes include intraoperative hypotension, embolism and interruption of the collateral circulation from the iliolumbar and internal iliac arteries [64].

## Conclusion

EVAR complications are common and can be life-threatening if not early identified by the radiologist on CTA and promptly treated. They can usually be eligible for an endovascular treatment. EVAR complications can be classified by time of onset, cause and severity. Careful detection and precise description of image findings are essential prerequisites for treatment success. CTA is currently the gold standard diagnostic technique used for the assessment of EVAR. In future perspectives, further possible new technologies (post-elaboration techniques with DECT acquisition, DSA, MRI and CEUS) can help where critical issues still arise.

## Data Availability

All data are included in this manuscript.

## Abbreviations

AAAs: Abdominal aortic aneurysms
CA: Celiac artery
CEUS: Contrast-enhanced ultrasound
CT: Computer tomography
CTA: Computer tomography angiography
CVR: Curvilinear reformation
DECT: Dual-energy computer tomography
DSA: Digital subtraction angiography
EL: Endoleak
ePTFE: Polytetrafluoroethylene
EVAR: Endovascular aneurysm repair
f/b EVAR: Fenestrated and branched endovascular aneurysm repair
MIP: Maximum intensity projection
MRA: Magnetic resonance angiography
NECT: Non-enhanced computer tomography
OR: Open repair
RRA: Right renal artery
SMA: Superior mesenteric artery
TOF-MRA: Time-of-flight magnetic resonance angiography
VR: Volume rendering
